# Buried PE Pipeline Location Method Based on Double-Tree Complex Wavelet Cross-Correlation Delay

**DOI:** 10.3390/s24227310

**Published:** 2024-11-15

**Authors:** Yang Li, Hanyu Zhang, Zhuo Xu, Ao Zhang, Xianfa Liu, Pengyao Sun, Xianchao Sun

**Affiliations:** 1 School of Mechanical Engineering, Northeast Electric Power University, Jilin City 132011, China; liyang891209@126.com (Y.L.); 13760944553@163.com (H.Z.); 2 School of Mechanical Engineering, Shenyang Jianzhu University, Shenyang 110168, China; zhangao2902@sjzu.edu.cn; 3 Hunan Angang Inspection and Testing Company, Changde 415131, China; 19974019370@163.com; 4 Special Equipment Inspection Center of Jilin (Special Equipment Accident Investigation Service Center of Jilin), Jilin City 132089, China; sageroc@163.com; 5 Changchun Special Equipment Inspection & Research Institute (Changchun Special Equipment Safety Monitoring Center), Changchun 130013, China; sunxc@cctj.freeqiye.com

**Keywords:** double-tree complex wavelet denoising, cross-correlation delay positioning, COMSOL simulated acoustic detection, buried PE pipeline

## Abstract

This study presents a location method for buried polyethylene (PE) pipelines based on the double-tree complex wavelet cross-correlation delay. Initially, the dual-tree complex wavelet transform (DTCWT) is applied to denoise the acquired signal, followed by extracting the delay time through the cross-correlation function to locate the buried pipeline. A simulation model is established to analyze the peak values of the time-domain signals in both asymmetric and symmetric sensor layouts using COMSOL, determining the relationship between the signal time differences and pipeline positions. Then, an experimental test system is set up, and experiments are carried out under the conditions of asymmetric and symmetrical sensors and different excitation points. The results indicate that the maximum error is 4.6% for asymmetric arrangements and less than 1% for symmetric arrangements. In practical applications, the pipeline’s position can be inferred from the delay time, with higher accuracy observed as the excitation point approaches the sensor. This method addresses the limitations of existing pipeline locating techniques and provides a foundation for the development of pipeline positioning technology.

## 1. Introduction

The underground pipeline system is an important part of urban infrastructure [1,2,3]. Due to their advantages of low costs and corrosion resistance, non-metallic pipelines have been used in the construction of underground pipeline networks. Among them, the PE pipeline is the most widely used in urban gas pipeline construction [4,5]. The pipeline exists in the soil environment for a long time, and the buried pipeline system is very prone to aging, cracking and other damage [6,7,8]. At the same time, it is difficult to determine the exact laying route and location for some buried pipelines with a long laying time. To ensure the safety of the urban underground pipe network, it is crucial to develop an efficient method to accurately locate buried PE pipelines.

In the buried pipeline, the leakage problem often produces unpredictable effects. Taking gas pipelines as an example, leakage will cause greater economic losses and even casualties. To avoid the loss of pipelines due to leakage, it is necessary to efficiently and accurately monitor the leakage point of the buried pipeline. In the context of monitoring and locating the leakage points of buried pipelines, relevant scholars have conducted some research and obtained promising results. Liang et al. [9] proposed a detection technology for the detection of leakage points. By collecting vibration signals caused by sound fields, this method can improve the detection ability for small leakages with a low false positive rate. Ozevin et al. [10] proposed a new leak location method by using two sensors to detect leaks at a single point. This method can accurately locate the leakage location and provides a theoretical basis for the realization of remote pipe network single-point wireless acoustic emission sensing. Cui et al. [11] proposed a technology to effectively locate leaks in gas pipelines by using a low-frequency narrowband acoustic emission method. The detection system can control the location errors of pipeline leakage points within 5%. Xu et al. [12] proposed a leak location method by using a multilevel framework. Experiments were carried out on buried pipelines with continuous leakage sources. The maximum error of the method was 5.3% regarding the location of the leakage point region when the sensor distance range was 10~33 m. Lang et al. [13] proposed a method for the location of pipeline leakage points by using information fusion; they combined ultrasonic sound velocity signals with flow signals to quickly and effectively locate small leaks. Mahmutoglu et al. [14] proposed a novel system based on passive acoustics. Based on the ambient noise, detection method, number of receivers, source strength, and number of measurements, this method can locate the leak point with a low average position error over a range of several kilometers. Xiao et al. [15,16] proposed a leak detection method for acoustic signals based on a wavelet transform and support vector machine (SVM). Building upon these previous studies, a correlation function model for gas pipeline leakage noise was established. The optimization of the correlation method in the detection and localization of gas pipeline leaks provides both theoretical and experimental support for gas pipeline leakage detection and localization. Ni et al. [17] studied the method of determining the specific leak location of the pipeline by using wavelet analysis to capture and analyze the drop points of pressure waves. This method can detect the exact location of the pipeline leakage point. Li et al. [18] considered specific application scenarios and studied various factors that affect field measurements, including environmental noise, welds, anti-corrosion coatings, and polyethylene coatings. The leakage point of the pipeline was analyzed by using a discrete wavelet transform and time spectrum. Zheng et al. [19] proposed a method to identify the leakage points of gas pipelines by collecting leakage noise in the soil. In the experimental scenario, the error when locating the leakage point of the buried gas pipeline was between 8% and 12%. Liu et al. [20] studied the propagation characteristics of negative pressure waves in branch pipe networks by taking the delay time of the pressure change as an indicator, and they studied the location of leakage points in heating pipelines by using the negative pressure wave method. The results showed that the method could locate the leakage point of the pipeline effectively. Most of the above studies have detected and analyzed the locations of pipeline leakage points when the locations of pipelines are known. However, if the locations of pipelines are unknown, the location of pipeline leakage points is prone to large errors. Therefore, if the location of the pipeline is unknown, it is necessary to first locate the pipeline.

To date, some scholars have conducted related research and obtained effective location methods for buried pipelines. Dai et al. [21] tested the location of underground pipelines through an acoustic signal detection system. This method can not only calculate the sound velocity of the soil but also determine the location of the underground pipeline. However, this method is difficult to operate in the process of pipeline positioning, and the positioning accuracy is low. Li et al. [22] proposed a method for the location of buried pipelines by using a combination of inclined angles and downward continuation. The position and depth information of the buried pipe could be calculated from the horizontal and vertical magnetic field gradients. This method is achieved using a vertical magnetic field gradient, but it is difficult to ensure the accuracy of pipeline positioning, since the magnetic field will be affected by environmental factors. Dong et al. [23] summarized 12 pipeline positioning methods and their advantages and disadvantages. On this basis, a new combination positioning method was proposed. This proposed method is suitable for urban PE gas pipe networks with complex and changeable environments. However, this method has the disadvantages of high costs, difficult operation, and low precision. Geng et al. [24] proposed a gas pipeline positioning method by using multi-frequency acoustic detection technology, which had incomparable advantages over the traditional positioning technology, especially for underground PE pipeline detection and positioning. However, due to the lack of noise reduction for the received signal, the positioning accuracy of the pipeline is difficult to ensure. Ge et al. [25] proposed a theoretical model of elastic wave propagation in a soil–PE pipeline medium based on the elastic wave reflection principle and proposed a new positioning method for buried PE pipelines based on time-domain superposition. The positioning accuracy achieved by this method could meet the needs of the on-site positioning of buried PE pipelines. These research results can be used to guide the research and development of acoustic buried PE pipeline detection and positioning equipment. However, when using these methods, the sound waves propagating in the soil will be affected by clutter waves. It can be seen from the above research that the current positioning methods for buried PE pipelines still need to be improved.

It is evident that research on the localization of buried pipelines in non-leakage conditions is relatively limited, and the existing localization methods still exhibit significant errors and poor accuracy. These methods usually are difficult to operate and come with high costs, making their widespread adoption challenging. Therefore, there is an urgent need for a localization method that offers higher accuracy, ease of operation, and a lower cost to accurately locate buried pipelines. Drawing on the research methods used to locate pipeline leakage points, this paper proposes a localization method based on the dual-tree complex wavelet cross-correlation time delay. This method integrates DTCWT denoising with cross-correlation function techniques to extract the signal delay time. On this theoretical basis, we first conduct a simulation analysis using the COMSOL Multiphysics 6.1 software to examine the sensor placement under both asymmetric and symmetric conditions. Subsequently, an experimental testing system is constructed, where excitation experiments are performed at different locations on the pipeline, with the sensors placed in asymmetric and symmetric configurations. The theoretical, simulation, and experimental results are then compared for error analysis to validate the reliability of the proposed pipeline localization method. This study effectively addresses the shortcomings of existing localization methods, enhancing both the accuracy and efficiency of pipeline localization. The method proposed in this paper can determine the direction of a buried pipeline by sequentially locating individual points along its horizontal axis, providing a theoretical basis for the localization of buried PE pipelines.

## 2. Theory

**Figure 1:**
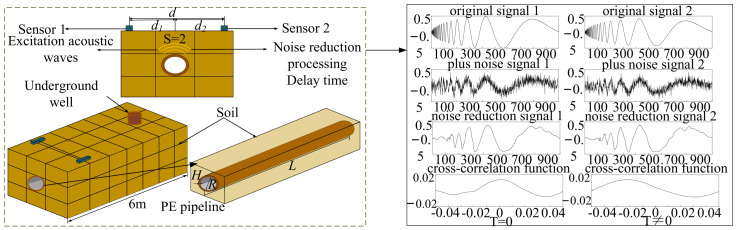
Positioning principle for buried PE gas pipeline.

In the context of DTCWT cross-correlation delay positioning, the main components include signal denoising and pipeline positioning. Figure 1 illustrates the principle of pipeline positioning, where the pipeline exposed in the underground well is excited. The vibration of the pipeline generates acoustic waves, which propagate through the soil and are detected by sensors arranged on the ground. The excitation frequency is set within the range of 0–15 kHz. When the excitation frequency is below the pipe ring frequency, typically, three axisymmetric modes are generated [26]: S = 1, primarily fluid-dominant waves; S = 2, mainly shell-dominant waves; S = 0, predominantly torsional waves. Among these, S = 0 represents torsional waves decoupled from the fluid inside the pipe, resulting in no radial vibration of the pipe wall. Therefore, the acoustic coupling between the gas inside the pipe and the pipe wall is manifested as S = 1 fluid-dominant waves and S = 2 shell-dominant waves. The displacement of the soil induced by shell-dominant wave radiation is greater than that induced by fluid-dominant waves. Therefore, effectively exciting shell-dominant waves facilitates the generation of stronger vibration signals and the acquisition of location information. Additionally, in the vibration model, the S = 2 wave corresponds to the longitudinal mode L(0,1) of the guided wave field within the column [27]. During signal acquisition, noise contamination can occur due to environmental factors and other interferences, necessitating the filtering and denoising of the acquired signals. Processing the denoised signals using cross-correlation functions allows for the determination of the delay time *T*_0_ between two sensors. When *T*_0_ = 0, it indicates that the pipeline is positioned at the midpoint between the two sensors.

After the excitation of the pipeline signal acquisition, the original signal is collected by the two sensors as the input signal $x_{1}(t)$, $x_{2}(t)$. For the input signal $x_{1}(t)$, $\psi_{h_{1}}(t)$ and $\psi_{g_{1}}(t)$ represent two real wavelets. Accordingly, their scaling functions are $\delta_{h_{1}}(t)$ and $\delta_{g_{1}}(t)$, respectively. The complex wavelet $\psi_{c_{1}}(t)$ can be expressed as

(1) $$\psi_{c_{1}}(t)=\psi_{h_{1}}(t)+i\psi_{g_{1}}(t)$$

where i is the imaginary unit.

The wavelet coefficient $d_{j_{1}}^{Re}$ of the real part tree and the coefficient $c_{J_{1}}^{Re}$ of the last level of the scale function can be calculated by the following formula:

(2) dj1Re(n)=2j2∫−∞+∞x1(t)ψh1(2jt−n)dt,j=1,2,3…JcJ1Re(n)=2J2∫−∞+∞x1(t)δh1(2Jt−n)dt

where *j*_1_ represents the number of decomposition layers (*j*_1_ = 1, 2, 3 … *J*_1_), and *J*_1_ is the maximum number of decomposition layers.

The wavelet coefficient $d_{j_{1}}^{Im}$ of the imaginary part tree and the coefficient $c_{J_{1}}^{Im}$ of the last level of the scale function can be calculated by the following formula:

(3) dj1Im(n)=2j2∫−∞+∞x1(t)ψg1(2jt−n)dt,j=1,2,3…JcJ1Im(n)=2J2∫−∞+∞x1(t)δg1(2Jt−n)dt

Through Equations (2) and (3), the complex wavelet coefficients $d_{j_{1}}^{C}(n)$ and the last scale function coefficient $c_{J_{1}}^{C}(n)$ of each layer are obtained as follows:

(4) dj1C(n)=dj1Re(n)+jdj1Im(n)cJ1C(n)=CJ1Re(n)+jCJIm(n)

Reconstructing the complex wavelet coefficients and the final level scaling function coefficients, the detail signals $D_{j_{1}}(t)$ at each level and the final approximation signal $C_{J_{1}}(t)$ can be derived:

(5) Dj1(t)=2j−12∑n=−∞+∞dj1Re(n)ψh1(2jt−n)+∑n=−∞+∞dj1Im(n)ψg1(2jt−n)

(6) CJ1(t)=2J−12∑p=−∞+∞CJ1Re(n)δh1(2Jt−n)+∑q=−∞+∞CJ1Im(n)δg1(2Jt−n)

The reconstructed signal ${\tilde{x}}_{1}(t)$ is equal to the sum of all detail signals and the approximation signal:

(7) $${\tilde{x}}_{1}(t)=C_{J_{1}}(t)+D_{j_{1}}(t),j=1,2,3\ldots J$$

Similarly, the reconstructed signal ${\tilde{x}}_{2}(t)$ is

(8) $${\tilde{x}}_{2}(t)=C_{J_{2}}(t)+D_{j_{2}}(t),j=1,2,3\ldots J$$

The two signals ${\tilde{x}}_{1}\left(t\right)$ and ${\tilde{x}}_{2}\left(t\right)$ are, respectively, replaced by *x*_1_(*x*, *t*) and *x*_2_(*x*, *t*), and the time delay *T*_0_ can be estimated by the peak of the cross-correlation function between the two signals, where the cross-correlation function $R_{{\tilde{x}}_{1}{\tilde{x}}_{2}}(\tau )$ can be expressed as

(9) $$R_{{\tilde{x}}_{1}{\tilde{x}}_{2}}(\tau_{0})=E\left[x_{1}(x_{1},t)x_{2}(x_{2},t+\tau_{0})\right]$$

where *E*[ ] denotes the expectation operator.

The cross-correlation coefficient $\rho_{{\tilde{x}}_{1}{\tilde{x}}_{2}}(\tau )$ is given by the following equation:

(10) $$\rho_{{\tilde{x}}_{1}{\tilde{x}}_{2}}(\tau_{0})=\frac{R_{{\tilde{x}}_{1}{\tilde{x}}_{2}}(\tau_{0})}{\sqrt{R_{{\tilde{x}}_{1}{\tilde{x}}_{1}}(0)R_{{\tilde{x}}_{2}{\tilde{x}}_{2}}(0)}}$$

In this equation, $R_{{\tilde{x}}_{1}{\tilde{x}}_{1}}(0)$ and $R_{{\tilde{x}}_{2}{\tilde{x}}_{2}}(0)$ are the auto-correlation functions of the total noise $x_{1}(x,t)$ and $x_{2}(x,t)$ at location τ=0.

The sound pressure s(x,ω) at frequency ω and distance *x* from the excitation point can be expressed in the following form:

(11) $$s(x,\omega )=s_{0}(\omega )e^{-ikx}$$

where $s_{0}(\omega )$ is the spectrum at *x* = 0, $k=\frac{\omega }{c}-i\zeta$ is the complex wavenumber with its real part related to the propagation wave speed, and the imaginary part represents the decay rate ζ of the sound pressure in the pipe due to losses. Furthermore, $\zeta =\zeta_{wall}+\zeta_{cl}$, where the pipe attenuation rate $\zeta_{wall}$ and the wave attenuation rate $\zeta_{cl}$ caused by viscous heating absorption in the fluid can be expressed as follows:

(12) $$\zeta_{wall}=\frac{1}{Rc}\sqrt{\frac{\upsilon \omega }{2}}+\frac{\gamma -1}{Rc}\sqrt{\frac{\chi \omega }{2}}$$

(13) $$\zeta_{cl}=\frac{\omega^{2}\mu }{2\rho_{f}c^{3}}\left(\left(\frac{4}{3}+\frac{\gamma -1}{\mathrm{Pr}}\right)\right)$$

In this equation, υ represents the dynamic viscosity, γ is the specific heat ratio, χ is the thermal diffusivity, *R* is the outer diameter of the pipe, and *c* is the velocity of the propagating acoustic wave along the gas pipeline. μ is the kinematic viscosity, $\rho_{f}$ is the fluid density, $\mathrm{Pr}=\frac{c_{p}\mu }{\kappa }$ is the Prandtl number, κ is the thermal conductivity, and $c_{p}$ is the specific heat capacity at constant pressure per unit mass.

Compared to the losses at the pipe wall, the losses within the fluid are much smaller; therefore, the wave attenuation rate $\zeta_{cl}$ can be neglected. Based on the above results, the complex wavenumber k of the propagating acoustic wave is obtained:

(14) $$k\approx \frac{\omega }{c}-i\left(\frac{1}{Rc}\sqrt{\frac{\upsilon }{2}}+\frac{\gamma -1}{Rc}\sqrt{\frac{\chi }{2}}\right)\sqrt{\omega }$$

Based on Equations (11) and (14), the frequency response function $H\left(\omega ,x\right)$ can be obtained.

(15) $$H\left(\omega ,x\right)=\frac{s\left(x,\omega \right)}{s_{0}\left(\omega \right)}=e^{-ikx}=e^{-i\frac{\omega }{c}x}e^{-\alpha \sqrt{\omega }x}$$

In this equation, $\alpha =\sqrt{\upsilon /2}/Rc+\left(\gamma -1\right)\sqrt{\chi /2}/Rc$.

The cross-spectral density $S_{{\tilde{x}}_{1}{\tilde{x}}_{2}}(\omega )$ between signals $x_{1}(x_{1},t)$ and $x_{2}(x_{2},t)$ can be expressed in the following form:

(16) $$\begin{array}{cc} S_{{\tilde{x}}_{1}{\tilde{x}}_{2}}(\omega ) & =\frac{1}{2\pi }\underset{T\to \infty }{\lim}E\left[\frac{s_{1T}^{*}(x_{1},\omega )s_{1T}(x_{2},\omega )}{T}\right]\\ & =S_{0}\left(\omega \right)\psi \left(\omega \right)e^{i\omega T_{0}} \end{array}$$

where $\Psi \left(\omega \right)=\left|H_{1}^{*}\left(\omega ,d_{1}\right)H_{2}\left(\omega ,d_{2}\right)\right|=e^{-\alpha \sqrt{\omega }\left(d_{1}+d_{2}\right)}$; $S_{0}\left(\omega \right)=1/2\pi \left(\underset{T\to \infty }{\lim}E\left[s_{0}^{*}\left(\omega \right)s_{0}\left(\omega \right)/T\right]\right)$ is the noise spectrum at *x* = 0; and $T_{0}=\frac{-\left(d_{2}-d_{1}\right)}{c}$ is the time delay.

We assume that the noise spectrum *S*_0_(ω) has more physical forms to predict the correlation function. The empirically measured cross-spectral density fits well with the following expression:

(17) $$S_{{\tilde{x}}_{1}{\tilde{x}}_{2}}(\omega )=\frac{8\rho_{f}^{2}c^{2}{\bar{u}}^{2}}{\pi^{4}}{\left(\frac{a}{R}\right)}^{4}\frac{\Lambda }{U}\frac{e^{-a\sqrt{\omega }(d_{1}+d_{2})}}{1+{\left(\frac{\omega \Lambda }{U}\right)}^{2}}e^{i\omega T_{0}}$$

The multiplication in the frequency domain corresponds to convolution in the time domain; therefore, the cross-correlation function can be expressed by the following equation:

(18) $$R_{{\tilde{x}}_{1}{\tilde{x}}_{2}}(\tau_{0})=\frac{8\rho_{f}^{2}c^{2}{\bar{u}}^{2}}{\pi^{4}}{\left(\frac{a}{R}\right)}^{4}\frac{\Lambda }{U}F^{-1}\left\{\frac{e^{-a\sqrt{\omega }(d_{1}+d_{2})}}{1+{\left(\frac{\omega \Lambda }{U}\right)}^{2}}\right\}⊗\delta \left(\tau_{0}+\tau \right)$$

where $F^{-1}\left\{\;\right\}$ denotes the inverse Fourier transform, and ⊗ represents the convolution operator.

Expressing Equation (18) in dimensionless form, the cross-correlation function can be represented as

(19) $$R_{{\tilde{x}}_{1}{\tilde{x}}_{2}}({\tilde{\tau }}_{0})=\frac{16\rho_{f}^{2}c^{2}{\bar{u}}^{2}}{\pi^{4}}{\left(\frac{a}{R}\right)}^{4}\int_{0}^{\infty }\frac{e^{-\sqrt{\Omega }({\tilde{d}}_{1}+{\tilde{d}}_{2})}e^{i\sqrt{\Omega }({\tilde{\tau }}_{0}+\tilde{\tau })}}{1+\Omega^{2}}d\Omega$$

where Ω=ωΛ/U is the dimensionless frequency, $\tilde{\tau }=\tau U/\Lambda$ is the dimensionless time lag, ${\tilde{T}}_{0}=T_{0}U/\Lambda$ is the dimensionless time delay, and ${\tilde{d}}_{i}$ is the dimensionless distance. ${\tilde{d}}_{i}$ can be expressed as

(20) $${\tilde{d}}_{i}=d_{i}\alpha \sqrt{\frac{U}{\Lambda }}$$

By employing a similar method, the cross-correlation function of $x_{1}(x,t)$ and $x_{2}(x,t)$ can be obtained, thereby deriving the cross-correlation coefficient:

(21) $$\rho_{{\tilde{x}}_{1}{\tilde{x}}_{2}}({\tilde{\tau }}_{0})=\frac{\int_{0}^{\infty }\frac{e^{-\sqrt{\Omega }({\tilde{d}}_{1}+{\tilde{d}}_{2})}e^{i\sqrt{\Omega }({\tilde{\tau }}_{0}+\tilde{\tau })}}{1+\Omega^{2}}d\Omega }{\sqrt{\int_{0}^{\infty }\frac{e^{-2\sqrt{\Omega }{\tilde{d}}_{1}}}{1+\Omega^{2}}d\Omega \cdot \int_{0}^{\infty }\frac{e^{-2\sqrt{\Omega }{\tilde{d}}_{2}}}{1+\Omega^{2}}d\Omega }}$$

When $\tilde{\tau }$ = −${\tilde{T}}_{0}$, the value of the cross-correlation coefficient is at its peak, with the peak expressed as

(22) $$\rho_{{\tilde{x}}_{1}{\tilde{x}}_{2}}(-\tilde{\tau })=\frac{\int_{0}^{\infty }\frac{e^{-\sqrt{\Omega }({\tilde{d}}_{1}+{\tilde{d}}_{2})}}{1+\Omega^{2}}d\Omega }{\sqrt{\int_{0}^{\infty }\frac{e^{-2\sqrt{\Omega }{\tilde{d}}_{1}}}{1+\Omega^{2}}d\Omega \cdot \int_{0}^{\infty }\frac{e^{-2\sqrt{\Omega }{\tilde{d}}_{2}}}{1+\Omega^{2}}d\Omega }}$$

Based on the above theory, the time delay *T*_0_ can be obtained using the computation of the cross-correlation function coefficient between two signals—that is, the time corresponding to the peak of the cross-correlation coefficient. When *T*_0_ = 0, it can be determined that the pipeline is positioned at the midpoint between the two sensors.

Once the time delay *T*_0_ is determined, the position *d*_1_ of sensor 1 relative to the center of the pipeline can be calculated:

(23) $$d_{1}=\frac{d-cT_{0}}{2}$$

According to this formula, the error of the pipeline positioning method based on the time delay can be verified.

## 3. Simulation Analysis

**Figure 2:**
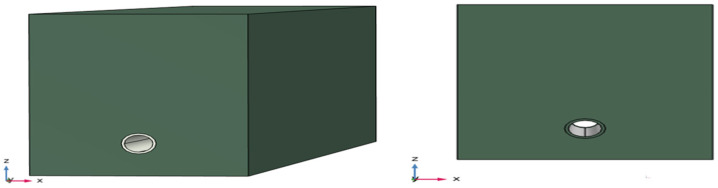
COMSOL simulation model.

COMSOL is used to verify the proposed method of locating pipelines according to the delay time *T*_0_. First, a pipeline model with a buried depth of 1 m, *L* = 6 m, *R* = 0.16 m, *H* = 0.0146 m PE is established in COMSOL, as shown in Figure 2. The material parameters of the pipeline and soil are shown in Table 1. The simulation of an asymmetric sensor arrangement and symmetrical sensor arrangement is carried out on the model to observe whether there is a time difference corresponding to the time-domain signal peak received by the two sensors—that is, the delay time *T*_0_.

**Figure 3:**
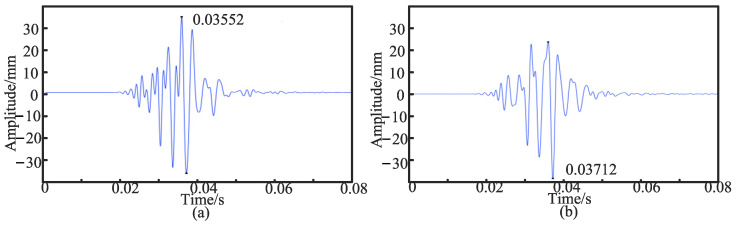
(**a**) Time-domain signal collected by the sensor at a position of 10 cm. (**b**) Time-domain signal collected by the sensor at a position of 50 cm.

First, the sensors were placed *d*_1_ = 10 cm and *d*_2_ = 50 cm away from the middle of the PE pipeline, and the time-domain signals of the two sensors were extracted. In Figure 3, Figure 3a is the time-domain signal collected by the sensor at a position of 10 cm, and its peak time *T*_1_ is 0.03552 s. Figure 3b shows the time-domain signal collected by the sensor at a position of 50 cm, and its peak time *T*_2_ is 0.03592 s.

For the asymmetric arrangement, other cases are also studied. The sensors are placed in the middle of the PE pipeline at positions *d*_1_ = 10 cm, *d*_2_ = 40 cm, *d*_1_ = 10 cm, and *d*_2_ = 30 cm to extract the time-domain signals of the two sensors, and the peak time obtained is shown in Table 2. It can be seen that when the sensors are asymmetrically arranged, there is a time difference between the time-domain signals received by the two sensors, and the time difference increases with the increase in the distance between the sensors.

**Figure 4:**
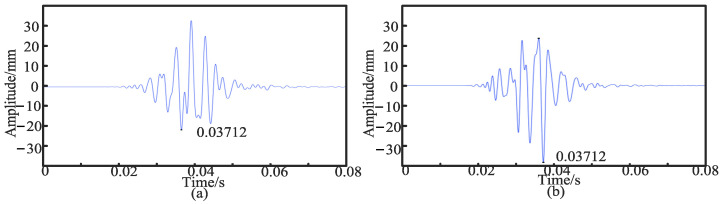
(**a**) Time-domain signal collected by sensor at 50 cm position. (**b**) Time-domain signal collected by sensor at 50 cm position.

Then, the simulation of the symmetrical sensor layout is carried out. The sensors are placed *d*_1_ = 50 cm and *d*_2_ = 50 cm away from the middle of the PE pipeline, and the time-domain signals of the two sensors are extracted. In Figure 4, Figure 4a is the time-domain signal collected by the sensor at a position of 50 cm, and its peak time *T*_1_ is 0.03712 s. Figure 4b shows the time-domain signal collected by the sensor at a position of 50 cm, and its peak time *T*_2_ is 0.03712 s.

In the other symmetrical arrangements, the sensor is placed in the middle of the PE pipeline at *d*_1_ = 40 cm, *d*_2_ = 40 cm, *d*_1_ = 30 cm, and *d*_2_ = 30 cm to extract the time-domain signals of the two sensors, and the peak time obtained is shown in Table 3. It can be seen that when the sensors are arranged symmetrically, the time of the signal received by the two sensors is equal, which means that the time difference is zero.

From the simulation results, it can be concluded that the proposed positioning method based on the time difference, i.e., the delay time, is feasible.

## 4. Experimental Verification

**Figure 5:**
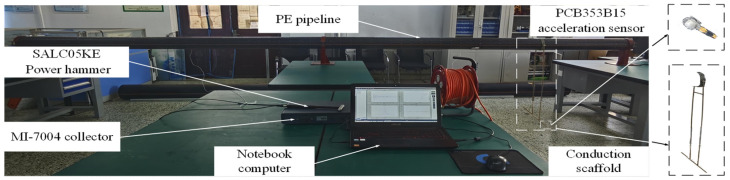
PE pipeline field experiment: device layout.

Based on the above theory and simulation, the pipeline experiment is carried out. As shown in Figure 5, the experimental equipment includes a MI-7004 signal collector (ECON TECHNOLOGIES CO., LTD., Hangzhou, China), a SALC05KE modal force hammer (Shiao Technology Co., Ltd., Wuxi, China), two PCB353B15 acceleration sensors, a computer, and a conduction bracket. The above equipment is used to simulate the positioning of a PE pipeline with *L* = 6 m, *R* = 0.16 m, and *H* = 0.0146 m in the soil environment. Table 4 shows the specific scheme of this experiment. The experiment includes eight groups, namely four groups with the asymmetric sensor arrangement and four groups with the symmetrical sensor arrangement, and the two sensors are placed on the conduction bracket.

To check the reliability of the data, each of the following group experiments is performed twice, extracting the two original signals received by sensor 1 and sensor 2. Using the double-tree complex wavelet theory to denoise the original signals, the correlation number of the two denoised signals can be obtained through the cross-correlation function, and the delay time *T*_0_ can also be obtained by analyzing the peak value of the correlation number.

**Figure 6:**
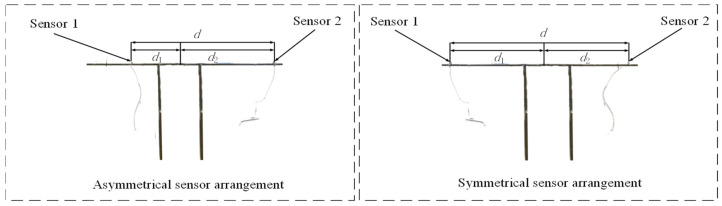
Location of the sensor in the conduction bracket.

**Figure 7:**
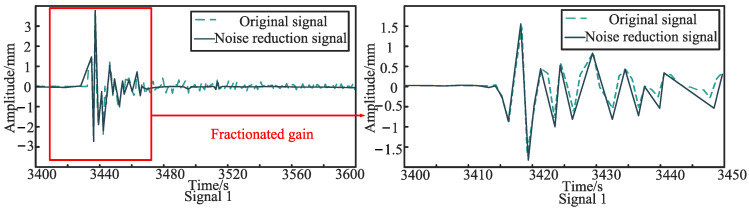
The original signal and noise reduction signal for the asymmetrically arranged signal 1 under the first excitation.

**Figure 8:**
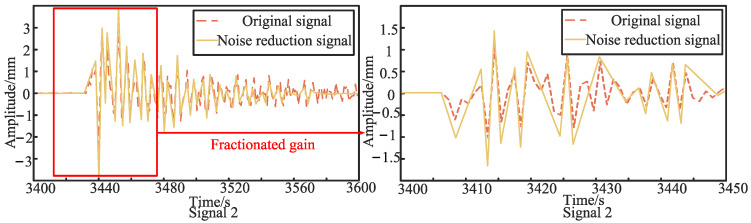
The original signal and noise reduction signal for the asymmetrically arranged signal 2 under the first excitation.

**Figure 9:**
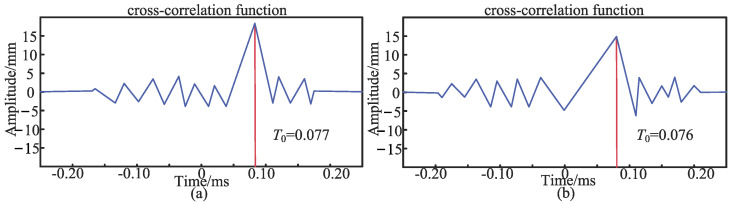
(**a**) The number of correlations for the asymmetric placement of the first excitation; (**b**) the number of correlations for the asymmetric placement of the second excitation.

The sensor layout is shown in Figure 6. Firstly, the first set of experiments on the asymmetric sensor arrangement is carried out. Sensor 1 and sensor 2 are asymmetrically arranged at positions *d*_1_ = 10 cm and *d*_2_ = 50 cm away from the middle of the PE pipeline, respectively, and the pipeline is excited at the position 2 m away from the left side of the conduction bracket. Figure 7 and Figure 8 show the original signals received by sensor 1 and sensor 2 under the first excitation and the signals after the noise reduction of the original signals. Taking the cross-correlation functions of signal 1 and signal 2, the number of mutual relations can be obtained, as shown in Figure 9a. According to the above theoretical analysis, the time at which the largest peak value of the mutual relation number occurs indicates the delay time *T*_0_ of the two signals. As can be seen from Figure 9a, under the first excitation, the delay time *T*_0_ of the two signals is 0.077 ms. For the second excitation, the processing of the signals is the same as for the first excitation and will not be presented again. The delay time *T*_0_ of the two signals in the second excitation is 0.076 ms, as seen in Figure 9b.

The second, third, and fourth sets of experiments are conducted with asymmetrical sensor placement. Two signals are obtained, and the delay time *T*_0_ is extracted. The delay time *T*_0_ is then substituted into Equation (23) to calculate the value *d*_0_, representing the distance from sensor 2 to the top of the pipeline. The experimental distance *d*_0_ is compared with the theoretical distance *d*_2_, and the error analysis results are shown in Table 5. It is evident that, with asymmetrical sensor placement, the cross-correlation of signals 1 and 2 received by sensors 1 and 2 results in a delay time that is significantly greater than zero. In this study, with asymmetrical sensor placement, the maximum error between the theoretical and experimental distances obtained by the two sensors is 4.6%. Furthermore, when the sensor placement is fixed, the experimental error increases as the excitation point moves farther from the sensor position. Therefore, the distance between the excitation points and the sensor placement also affects the delay time *T*_0_. At this stage, the sensor placement simulates the scenario whereby the pipeline is not centered between the two sensors during actual detection. In this case, further detection is required to accurately locate the pipeline.

**Figure 10:**
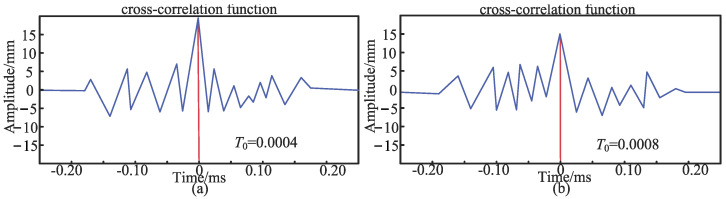
(**a**) The number of correlations for the asymmetric placement of the first excitation; (**b**) the number of correlations for the asymmetric placement of the second excitation.

After the asymmetric test, the first set of experiments on the symmetrical sensor arrangement is carried out. Sensors 1 and 2 are placed in symmetrical positions *d*_1_ = 50 cm and *d*_2_ = 50 cm away from the middle of the pipeline. The pipe is excited 2 m from the left side of the conduction bracket. As shown in Figure 10, the relationship between the two signals is *T*_0_. Under the first excitation, the delay time *T*_0_ of the two signals is 0.0004 ms. Meanwhile, under the second excitation, the delay time *T*_0_ of the two signals is 0.0008 ms.

Subsequently, the second, third, and fourth sets of experiments are conducted with symmetrical sensor placement. The delay time *T*_0_ obtained from the experiments is substituted into Equation (23) to calculate the experimental distance *d*_0_ from sensor 2 to the top of the pipeline. The experimental distance *d*_0_ is then compared with the theoretical distance *d*_2_, with the results shown in Table 6. It is evident that, with symmetrical sensor placement, the cross-correlation of signals 1 and 2 received by sensors 1 and 2 results in a delay time close to zero. In the experimental results with symmetrical sensor placement, the maximum error between the experimental distance calculated from the delay time *T*_0_ and the theoretical distance is only 0.9%. When the sensor placement is fixed, the experimental error increases as the excitation point moves farther from the sensor position. Therefore, the distance between the excitation points and the sensor position also affects the delay time *T*_0_.

The symmetrical sensor placement simulates the scenario whereby the pipeline is located at the midpoint between the two sensors during actual detection. Asymmetrical sensor placement simulates the condition in which *T*_0_ ≠ 0, indicating that the pipeline is not centered between the two sensors, and requires further sensor adjustment for localization. When the sensors are symmetrically placed relative to the pipeline, the arrival times of the two signals are nearly equal. Thus, when *T*_0_ = 0, it can be inferred that the pipeline is located approximately midway between sensors 1 and 2. In practical engineering, due to inevitable errors, if the error between the experimental and theoretical distances is within 1%, the pipeline can be considered to be near the midpoint between the two sensors, enabling the more accurate localization of the pipeline’s position.

### Comparison with Other Pipeline Localization Methods

To further validate the method proposed in this study, we compare the experimental error results with those of pipeline localization methods from other research. Zhou et al. [28] proposed a new model that estimated the pipeline’s orientation and radius from GPR B-scan images based on the dielectric constant of an underground medium. A comparison of the model results with the actual results is shown in Table 7. It can be seen that the maximum error in locating the buried pipeline orientation using Zhou’s method is 8.4%, while the maximum error with our method is only 4.6%. Therefore, this method significantly improves the localization accuracy compared to the GPR-based method when determining the pipeline orientation. Additionally, it is more convenient to operate and more cost-effective than other methods, providing a feasible approach to detecting the positions of buried PE pipelines.

## 5. Conclusions

This paper presents a pipeline location method based on the double-tree complex wavelet cross-correlation delay. The signal is denoised via the double-tree complex wavelet method, and the delay time of the denoised signal is obtained with a cross-correlation function to determine the location of the buried pipeline. To verify the reliability of the positioning method proposed in this paper, different pipeline positions are excited considering the asymmetric and symmetrical layout of the sensors in the experiment. Multiple sets of acquisition signals are obtained, and the delay time between the sensors is obtained for each set, leading to the realization of pipeline positioning. The main conclusions of this paper can be summarized as follows.

(1) In the case of asymmetrical sensor placement, the delay time obtained from the cross-correlation of signals 1 and 2 is used to calculate the distance *d*_0_. The error compared to the theoretical distance *d*_2_ is significantly greater than 1%. In the case of symmetrical sensor placement, the delay time obtained from the cross-correlation of signals 1 and 2 results in an error of less than 1% when compared to the theoretical distance *d*_2_. Based on the above results, it can be inferred that the pipeline is located approximately in the middle of the two sensors when the error is less than 1%.

(2) Comparing the results of the method proposed in this study with those of other studies clearly shows that our method offers an improvement in the accuracy of buried pipeline localization. Additionally, it is found that the position of the excitation point relative to the sensors also affects the localization performance. In both asymmetrical and symmetrical sensor placement, the farther the excitation point is from the sensor position, the greater the localization error. This factor can influence the accuracy of pipeline localization to some extent in practical applications.

The method proposed in this paper can determine the locations of buried pipelines quickly and accurately, with simple operation and a low cost, having certain guiding significance for the location of buried PE pipelines.

## Figures and Tables

**Table 1:** Material parameters of pipe and soil.

Parameter		Pipeline	Soil
Density (kg/cm^3^)	ρ	950	2000
Young’s modulus (GPa)	*E*_1_	1.08	0.083
	*E*_2_	1.08	0.083
Shear elasticity (GPa)	*G*_12_	0.38	-
Poisson’s ratio	υ	0.418	0.3

**Table 2:** Asymmetric sensor arrangement time.

Simulation	Sensor 1 Position *d*_1_ (cm)	Sensor 2 Position *d*_2_ (cm)	*T*_1_	*T*_2_
Asymmetric arrangement	10	40	0.03552	0.03700
	10	30	0.03552	0.03692

**Table 3:** Time-domain signal collected by sensor at 50 cm position.

Simulation	Sensor 1 Position *d*_1_ (cm)	Sensor 2 Position *d*_2_ (cm)	*T*_1_	*T*_2_
Symmetric arrangement	40	40	0.03700	0.03700
	30	30	0.03692	0.03692

**Table 4:** Specific experimental plan.

Arrangement Form	Group	Sensor Location		Excitation Point Position (Distance from Conduction Bracket Position)
		*d*_1_ (cm)	*d*_2_ (cm)	
Asymmetric arrangement	The first group	10	50	2
	The second group	10	50	4
	The third group	10	30	2
	The fourth group	10	30	4
Symmetrical arrangement	The first group	50	50	2
	The second group	50	50	4
	The third group	30	30	2
	The fourth group	30	30	4

**Table 5:** The delay times in the four groups of experiments under the asymmetric sensor arrangement.

Number of Experiments	Sensor 1, 2 *d*_1_, *d*_2_/cm	Excitation Point Position/m	Experiment *T*_0_/ms	Experiment *d*_0_(*K*)/cm	Theory *d*_2_(*H*)/cm	Error (|*K* − *H*|/*K*)/%
First experiment	10, 50	2	0.077	50.51	50	1.0%
	10, 50	2	0.076	49.43	50	1.1%
Second experiment	10, 50	4	0.079	48.29	50	3.3%
	10, 50	4	0.078	51.59	50	3.1%
Third experiment	10, 30	2	0.038	30.39	30	1.3%
	10, 30	2	0.039	29.64	30	1.2%
Fourth experiment	10, 30	4	0.041	31.28	30	4.1%
	10, 30	4	0.040	31.45	30	4.6%

**Table 6:** Delay time and error for four groups of experiments under symmetrical sensor arrangement.

Number of Experiments	Sensor 1, 2 *d*_1_, *d*_2_/cm	Excitation Point Position/m	Experiment *T*_0_/ms	Experiment *d*_0_(*K*)/cm	Theory *d*_2_(*H*)/cm	Error (|*K* − *H*|/*K*)/%
First experiment	50, 50	2	0.0004	50.05	50	0.1%
	50, 50	2	0.0008	50.15	50	0.3%
Second experiment	50, 50	4	0.001	50.20	50	0.4%
	50, 50	4	0.002	49.75	50	0.5%
Third experiment	30, 30	2	0.0008	30.15	30	0.5%
	30, 30	2	0.0006	30.18	30	0.6%
Fourth experiment	30, 30	4	0.001	29.79	30	0.7%
	30, 30	4	0.0015	30.27	30	0.9%

**Table 7:** The error between the localization model results and the actual results of the pipeline [28].

Area	The Average Error		The Max Error	
	Directions (*α*)	Radius (*b*)	Directions (*α*)	Radius (*b*)
1	4.03%	5.23%	7.14%	7.22%
2	5.10%	5.90%	8.40%	7.41%

## Data Availability

Data will be provided upon request.
